# An Integrated Analysis of miRNAs and Methylated Genes Encoding mRNAs and lncRNAs in Sheep Breeds with Different Fecundity

**DOI:** 10.3389/fphys.2017.01049

**Published:** 2017-12-15

**Authors:** Xiangyang Miao, Qingmiao Luo, Huijing Zhao, Xiaoyu Qin

**Affiliations:** State Key Laboratory of Animal Nutrition, Institute of Animal Sciences, Chinese Academy of Agricultural Sciences, Beijing, China

**Keywords:** sheep, high fecundity, miRNA, methylation, lncRNA

## Abstract

In our previous study, we investigated the regulatory relationship between lncRNAs, miRNA, and mRNAs in an effort to shed light onto the regulatory mechanisms involved in sheep fecundity. As an extension of this study, here, we aimed to identify potential regulators of sheep fecundity using a genome-wide analysis of miRNAs and the methylated genes encoding mRNAs and lncRNAs in the ovaries of Dorset sheep (low fecundity) and Small Tail Han ewes (high fecundity) with the genotype BB (Han BB) and the genotype ++ (Han ++) by performing RNA-Seq and MeDIP-Seq analyses. Methylated coding-non-coding gene co-expression networks for Han and Dorset sheep were constructed using the methylated genes encoding the differentially expressed mRNAs and lncRNAs identified in this study. In the Han BB vs. Dorset comparison, the lncRNAs TTC26 and MYH15 had the largest degree. Similarly, the lncRNA NYAP1 had the largest degree in the Han ++ vs. Dorset comparison. None of the methylated genes encoding lncRNAs were co-expressed with the methylated genes encoding mRNAs in the Han BB vs. Han ++ comparison. The methylated genes encoding lncRNAs identified here may play a vital regulatory role in sheep breeding. Our results suggest that miRNAs might play a key role in sheep prolificacy by regulating target genes related to thyroid hormone synthesis, and methylated genes encoding lncRNAs associated with tight junctions might contribute to the high breeding rate in Han sheep. These findings may contribute to a deeper understanding of sheep prolificacy.

## Introduction

Sheep (Ovis aries) constitute a vastly diverse species that are reared for their meat, milk, fiber, and skin. One of the main goals in sheep breeding across the world is high prolificacy. However, most sheep species generate only one lamb, and just a few produce twin lambs, and as a result, the breeding production is greatly affected. Considering the low heritability of the sheep lambing number, it is difficult to change this feature through traditional selection. Accordingly, an increasing number of scientists are paying much more attention to the candidate genes and non-coding RNAs (ncRNAs) associated with fecundity (Miao and Luo, 2013; Mallory and Shkumatava, 2015; Miao et al., 2016a,c). High prolificacy is a complex trait that is affected by multiple factors, including age, season, genes (the most important factor) and nutrition (Michels et al., 2000; Rekiel et al., 2014). Therefore, it is difficult to identify the candidate genes or ncRNAs with a single molecular technique.

An excellent local breed in the Shandong province in north China is the Small Tail Han sheep (Han sheep), and in the United States, Dorset sheep are the most prevalent white-faced breed (Miao et al., 2015a,b, 2016b). Several fecundity genes in sheep have been identified to explain the underlying genetic mechanism, including growth and differentiation factor-9 (GDF9), bone morphogenetic protein-15 (BMP15) and BMP receptor-1B (BMPR1B) (Cemal et al., 1996; Fabre et al., 2006). Although existing genetic studies have already identified numerous sheep fecundity genes, the underlying genetic mechanism(s) remain largely undefined. A type of small non-coding RNA, known as microRNA (miRNA), plays a vital role in both plants and animals by regulating protein-coding genes (Miao et al., 2015a,b, 2016c,e; Guo et al., 2017; Miao, 2017). In addition, long non-coding RNAs (lncRNAs) have recently been characterized in animals because they regulate the expression of neighboring coding genes, which may be important for development (Lee, 2012). In addition to non-coding RNAs (miRNAs and lncRNAs), DNA methylation is related to epigenetic mechanisms. Importantly, epigenetic modifications, which are inherited alterations in gene expression that do not alter the DNA sequence, are being actively investigated in the field of sheep fecundity research (Goddard and Whitelaw, 2014; Gonzalez-Recio et al., 2015). However, there are few reports about the methylation of the genes encoding proteins or lncRNAs.

To investigate the potential role of miRNAs and the methylated genes that potentially encode lncRNAs in regulating sheep fecundity, we performed RNA-Seq analysis to identify genome-wide miRNAs and lncRNAs in two distinct breeds of sheep, Han (both the BB and ++ genotypes) and Dorset. An excellent local breed in the Shandong province in north China that is a rich source of meat is the Small Tail Han sheep (Han sheep). Dorset sheep, which originated in England and are the most prevalent white-faced breed in the United States, have a medium body size with robust muscle conformation (Miao et al., 2015a, 2016b). Even though Dorset sheep grows faster than Han sheep, Dorset sheep has a comparatively lower prolificacy (1.45 in Dorset vs. 2.61 in Han sheep) (Tu et al., 1989; Freking et al., 2000). These two breeds also differ in their fat deposition patterns (Miao et al., 2015c,d). Within the Han breed, Han BB genotype produce 1.4 more lambs more than the Han ++ sheep (Miao et al., 2015b, 2016b). We further constructed co-expression networks using the mRNA-miRNA and mRNA-lncRNA pairs that were associated with sheep breeding to examine the patterns of interaction among the genes with their associated co-expressed miRNAs and lncRNAs. Unlike previous studies, which focused only on the genes associated with sheep fecundity, we further identified methylated genes that encode mRNAs and lncRNAs that correspond to sheep proliferation, as well as the associations between mRNAs and lncRNAs in the three sheep groups. We rationalized that investigations of the patterns of genomic diversity in breeds with different prolificacy will provide genomic indicators associated with fecundity that can be confirmed in additional functional studies. Comparison of two different breeds is expected to reveal widespread genome wide changes. However, our design of including both BB and ++ genotype of Han and the Dorset allowed comparison both within the same breed and between different breeds with different prolificacy.

## Materials and methods

### Ethics statement

All of the animal procedures were approved by the Institutional Animal Care and Use Committee (IACUC) at the Institute of Animal Sciences, Chinese Academy of Agricultural Sciences, which is where the experiments were conducted. All methods were carried out in accordance with the relevant guidelines and regulations set by the Ministry of Agriculture of the People's Republic of China.

### Samples

Small Tail Han sheep (Han) and Dorset sheep (Dorset) were bred by the Ao-Te sheep breeding farm in Qingdao (Shandong, China). Blood samples were obtained from the Han ewes to identify the FecB mutation in the BMPR1B gene. Among these ewes, the high-fecundity Han sheep were the adult Han ewes with the genotype BB (HanBB) and the genotype ++ (Han ++), and they were regarded as the two groups. In addition, the adult Dorset ewes were sacrificed to obtain the ovary samples and were considered the low-fecundity group and served as controls. All of the animals were raised under similar conditions and had free access to water and food in natural lighting. Three animals from each group (Dorset, HanBB, and Han++) were used for the experiments described below.

All of the experimental ewes were administered with intravaginal sponges (40 mg, Chronogest, Intervet, Federal District, Mexico) that were impregnated with fluorogesterone acetate. Ten days later the sponges were removed, and then, the estrus synchronization, the ewes received an i.m. injection of pregnant mare serum gonadotropin (Ningbo HormoneCo., Ningbo, China) at a dose of 400 IU (Quintero-Elisea et al., 2011). Estrus was confirmed, and all of the sheep that reached spontaneous estrus after an estrous cycle were sacrificed between 24 and 36 h after the spontaneous estrus was detected. The animals were all between 2.5- and 3-year-old when they were euthanized for ovary harvesting. Whole ovaries were removed, and in order to obtain better ovulation points on the surface of the ovaries, samples were collected. All of the samples (3 animals per group) were immediately snap-frozen in liquid nitrogen and were stored at −80°C until the RNA isolation.

### RNA isolation and sequencing

The total RNA from the ovaries was isolated using the TRIzol reagent (Invitrogen Carlsbad, CA), and the RNA integrity number (RIN) for all of the samples was >8. An additional RNase-free DNase I (Ambion, Inc., Austin, TX, USA) digestion step was conducted to ensure that the samples were not contaminated with genomic DNA. After isolating the total RNA from the samples (3 Dorset, 3 Han BB and 3 Han ++), we constructed whole transcriptome libraries using TruSeq Stranded Total RNA with Ribo-Zero Globin (Illumina, San Diego, CA, USA) according to the manufacturer's instructions. The quality control and quantification of the libraries were performed using the BioAnalyzer 2100 system and qPCR (Kapa Biosystems, Woburn, MA, USA), respectively. Initially, the resulting libraries were sequenced on a HiSeq 2000 instrument, which produced 100 nucleotide paired-end reads. Additionally, small RNAs, between 18 and 30 nt long, were purified from the total RNA from each sheep group. Then, we constructed small RNA libraries that represented each species using the Illumina® TruSeq™ Small RNA Sample Preparation protocol. Briefly, the total RNA was ligated with an RNA 3′ adaptor and 5′ adaptor. Next, the RNA was reverse transcribed with SuperScriptII Reverse Transcriptase (Invitrogen). To obtain the miRNA library, the cDNA was amplified by PCR and was then purified. The Qubit™ dsDNA HS kit and a Qubit® 2.0 Fluorometer was used to assess the quality of the library. The size and purity of the library were assessed using High Sensitivity DNA Chips and the Agilent 2100 system. Clusters were generated using the cBot of Illumina Genome Analyzer IIx according to the manufacturer's instructions. Finally, the RNA libraries were sequenced using the single-read multiplex program in the Shanghai Biotechnology Corporation. The data collection software from Illumina was used to control the sequencing process.

### Transcriptome assembly and data analysis

Using the TopHat default settings, the RNA-Seq reads from each sample were aligned to the oar 3.1 sheep reference genome (Trapnell et al., 2009). The transcript annotation of the sheep genome sequence (Release number: Oar3.1) was downloaded (ftp://ftp.ncbi.nlm.nih.gov/genomes/Ovis_aries/).

The gene expression analysis only included the uniquely mapped reads. Based on the previously described rigorous significance test for digital gene expression, the significantly differentially expressed genes (DEGs) were identified using the DESeq package (Anders, 2010). The error rate adjustment in the multiple significance tests was assessed using the false discovery rate (FDR) (Benjamini and Hochberg, 1995). If the fold change was >1.5 or <0.667 and the FDR was <0.05, then the genes and the lncRNAs were considered to be differentially expressed.

### DNA preparation and MeDIP-Seq

Genomic DNA was isolated from each group (3 Han BB, 3 Han ++ and 3 Dorset ovaries) by a phenol-chloroform extraction method. Briefly, the DNA was sheared by sonication on ice to generate random fragments ranging from 100 to 500 bp. The sonicated DNA (5 μl) was used for the immunoprecipitation (IP). Next, the recovered DNA was 5′ and 3′ end blunted, phosphorylated and was repaired by T4 polynucleotide kinase and T4 DNA polymerase (NEB, USA). Then, the DNA was ligated to an Illumina sequencing primer after the addition of an ATP in the 3′ end. Our MeDIP method was the same as the one described by Zhang et al. (2006). After MeDIP, the DNA was PCR amplified with sequencing primers provided by Illumina, and the PCR products were recovered and used for sequencing.

### MeDIP-Seq sequence alignments and data analysis

We downloaded the sheep reference sequence and mapping information from the University of California Santa Cruz Genome Bioinformatics Site (http://genome.ucsc.edu). The sequencing reads and the resulting files were aligned to the sheep reference genome. Model-based analysis for ChIP-Seq (MACS) was applied for peak detection and analysis of the IP sequencing data.

### Functional annotation of the methylated DEGs

The identified methylated DEGs were analyzed with GO (Ashburner et al., 2000), which is an international standardized gene functional classification system. The DEGs were annotated by three structured controlled ontologies, including biological process (BP), molecular function (MF), and cellular component (CC), which were used to maintain a consistent description of the gene products. In addition, the Kyoto Encyclopedia of Genes and Genomes (KEGG) database was used to determine the association of the genes with different pathways (Kanehisa and Goto, 2000). To select the significant GO terms and pathways, a Fisher's exact test followed by the Benjamini-Hochberg (BH) multiple testing correction was performed to calculate the threshold of significance. The GO terms and pathways with a *p* < 0.05 were considered significant.

### Identification of miRNAs regulating sheep fecundity

The small RNA reads acquired by the Illumina Hiseq 2000 were subjected to several filtering processes, including low quality read filtering and removing the adaptor sequences. Finally, only short trimmed reads sized from 18 to 30 nt were retained. To annotate and classify the small RNAs into different categories and to identify novel miRNAs, the small RNA reads were compared with the known non-coding RNAs deposited in the Rfam database. The rest were subjected to RNAfold, which identifies miRNA candidates according to the hairpin secondary structure (Hofacker et al., 1994). The identified miRNAs were regarded as novel candidate miRNAs if they were absent in the miRBase database (Griffiths-Jones et al., 2006). To study the regulatory relationship between the mRNA and the miRNA, an mRNA-miRNA regulatory network was constructed and was displayed using Cytoscape software (Smoot et al., 2011).

### Construction of the co-expression network

To generate the co-expression network, methylated genes that encoded the DEGs and the differentially expressed lncRNAs were selected. Meanwhile, the differentially expressed lncRNAs with length of 200–800 bp were used to construct a network. The Pearson correlation was calculated for each pair of genes that were analyzed, and the pairs (only lncRNA-mRNA) with significant correlations were selected to construct the network. Only the strongest correlations (0.99 or greater) were included in the network to generate a visual representation. Cytoscape software was applied to draw the co-expression networks (Smoot et al., 2011). Finally, the degree of nodes in each network was calculated.

Meanwhile, a weighted gene co-expression network was constructed to identify the interactions among the methylated genes that encoded the DEGs and the differentially expressed lncRNAs by applying the WGCNA approach (Zhang and Horvath, 2005; Wang et al., 2013). The weighted Pearson correlation matrices, corresponding to the gene or lncRNA expression, were first calculated to generate the co-expression network, which was followed by the standard procedure of the WGCNA (Wang et al., 2009). Briefly, the weighted correlation matrices were transformed into connection strength matrices. Then, the connection strengths were used to calculate the topological overlap, which is a powerful and biologically meaningful measurement of the strength of the relationship of the co-expression of two genes with other genes in the network (Yip and Horvath, 2007). The genes that had highly similar co-expression relationships were grouped using hierarchical clustering based on the topological overlap. The Dynamic Hybrid Tree Cut algorithm was used to generate the hierarchal clustering tree, and the modules were defined as branches from the tree cutting (Langfelder et al., 2008). The minimum spanning tree with a dissimilarity matrix from the WGCNA was used to generate the network for each module. The modules were initially denoted by colors, and the gray color denoted the background genes outside of the modules. The highly correlated genes in each module were summarized by the first principal component, which was referred to as the module eigengene. Modules with a *p* < 0.1 were merged for further analyses. To identify the genes in the significant modules associated with proliferation, we calculated the intramodular connectivity (k) by summing the connection strengths with other module genes and dividing the number by the maximum intramodular connectivity. The genes with the highest intramodular connectivity were referred to as hub genes.

### Quantitative PCR (qPCR)

To validate the identified differentially expressed transcripts, miRNAs and lncRNAs in the three groups, we performed real-time PCR using SYBR Green I Master Mix in a Roche LightCycler 480 II Real Time PCR system. 5S rRNA (for miRNA) and 18S rRNA (for mRNA and lncRNA) were used as internal controls in these experiments. The PCR was performed using the following program: 95°C for 10 min; 40 cycles of 95°C for 15 s with a melting temperature for 30 s; and 72°C for 45 s; and 72°C for 5 min.

### Statistical analyses

The DEG data and the bioinformatics analyses are described above. The data from the qPCR are presented as the mean ± SD. Comparisons between the groups (the Han group vs. the Dorset group) from the qPCR were made using a Student's *t*-test, and *p* < 0.05 was considered statistically significant.

## Results

### Genome-wide profiling to identify the differentially expressed genes (DEGs) and lncRNAs

The mRNA libraries that were derived from the Han BB, Han ++ and Dorset sheep ovaries were sequenced and analyzed. Functional genetic differences among the three groups of sheep were assessed by identifying the DEGs in the Han ++ vs. Dorset, the Han BB vs. Dorset and the Han BB vs. Han ++ pairwise groups (Tables S1–S3). Compared with the Dorset group, 2,511 genes were differentially expressed in the Han BB group (Table S1), and 1,280 genes were differentially expressed in the Han ++ group (Table S2). Compared with the Han ++ group, 2,241 genes were differentially expressed in the Han BB group (Table S3). To identify candidate lncRNAs that might be related to the proliferation of sheep, we focused on the differentially expressed lncRNAs in the three comparison groups. Compared with the Dorset group, there were 138 lncRNAs that were differentially expressed in both the Han BB and ++ groups. There were 106 lncRNAs that were differentially expressed in the Han BB group compared with the Han ++ group.

### Global mapping of the DNA methylation in the three sheep groups

Next, we performed methylated DNA immunoprecipitation sequencing (MeDIP-Seq) with an Illumina Hiseq 2000 to reveal the genome-wide DNA methylome of the sheep. The MeDIP-Seq reads were aligned to a reference sequence, and for scanning the methylation peaks, we only used the unique mapped reads. A range of 112,892,114 to 114,963,972 raw reads was generated for the three sheep groups. More than 93% of the reads were mapped, and about 55% of the reads in each group were uniquely mapped to the sheep genome for each sample (Table 1).

**Table 1:** MeDIP-Seq Illumina data.

**Sample**	**Total reads**	**Mapped reads**	**Mapping rate (%)**	**Unique mapped reads**	**Unique mapping rate (%)**
Dorset	112,892,114	105,786,974	93.71	62,291,248	55.18
Han BB	114,963,972	107,924,232	93.88	64,171,269	55.82
Han ++	113,264,664	106,503,861	94.03	63,333,133	55.92

In addition, the distribution of the DNA methylation in the regions 2 kb upstream 2 kb downstream of the transcription termination sites, in the CpG island and in the intragenic region were analyzed. In general, higher levels of DNA methylation were revealed in the CpG islands and the gene body regions compared to the upstream and downstream regions. In sheep, the DNA methylation level increased sharply in the CpG islands and the intragenic region (Figure S1). To assess the effect of methylation on transcriptional activity and its relation to sheep reproduction, we analyzed the differentially methylated genes based on the peak coverage within the different elements in the three comparison groups (Figure S2). We found that the number of differentially methylated genes in the three comparison groups was similar.

### Integrated analysis of the MeDIP and gene expression data

To identify whether there was a relationship between the methylated genes encoding the DEGs and the differentially expressed lncRNAs, the expression data were compared with the MeDIP data. Compared with the Dorset group, among the DEGs and the differentially expressed lncRNAs, 73 were methylated in the Han BB group, and 129 were methylated in the Han ++ group. When compared with Han ++, there were only 7 methylated genes in the Han BB group.

### GO and KEGG pathway enrichment analyses of the methylated DEGs

**Figure 1:**
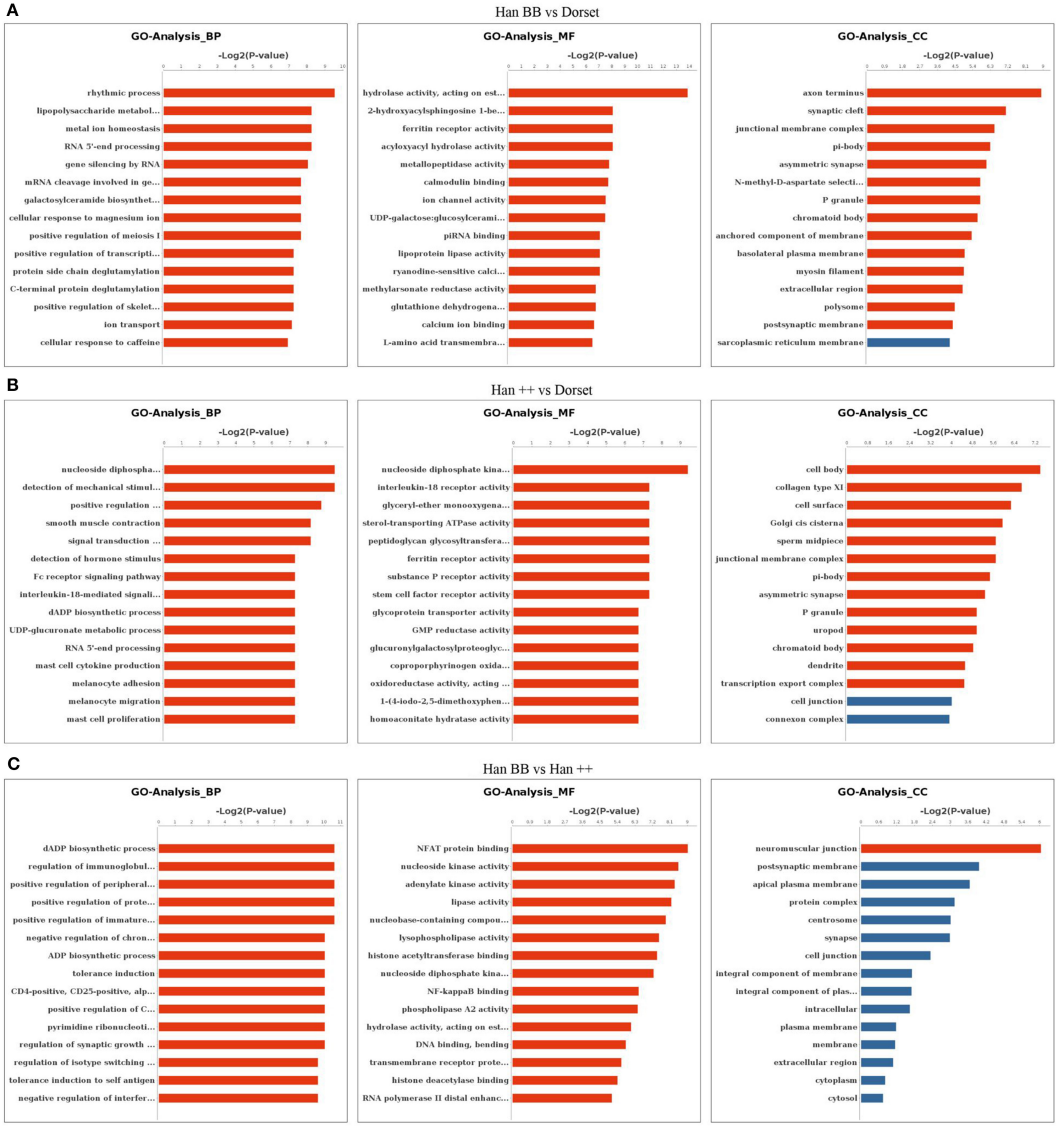
Gene Ontology analysis of the methylated differentially expressed genes in the different sheep groups. **(A)** Han BB vs. Dorset; **(B)** Han ++ vs. Dorset; **(C)** Han BB vs. Han ++. Han BB, Small Tail Han ewes with the genotype BB (HanBB) and genotype ++ (Han ++); Dorset, Dorset sheep. BP, Biological process; CC, cell component; MF, molecular function.

To highlight important biological processes related to the methylated DEGs among the three groups of sheep, we performed a GO enrichment analysis. The methylated DEGs in the Han BB group compared with Dorset were mainly associated with rhythmic process, lipopolysaccharide metabolic process and metal ion homeostasis (Figure 1A). A comparison of Han ++ group to the Dorset group revealed highly enriched GO terms that were related to development, cell differentiation and signal transduction (Figure 1B). A comparison between the Han BB and Han ++ groups revealed categories associated with biosynthetic process and immune response (Figure 1C). The GO terms for CC and MF were compared among the 3 sheep groups, and the results were similar (Figure 1).

To further explore the pathways enriched by the methylated DEGs, we carried out a KEGG enrichment analysis. A comparison of the genes in the Han BB group with those in the Dorset group, showed pathways that were significantly enriched in secretion, metabolism and protein functions (Table S4). When the Han ++ groups were compared to the Dorset group, the DEGs were mainly related to endocytosis, metabolism and secretion (Table S5). A comparison of the Han BB group with the Han ++ group revealed that the genes were associated with metabolism (Table S6).

### Identification of the differentially expressed miRNAs

**Figure 2:**
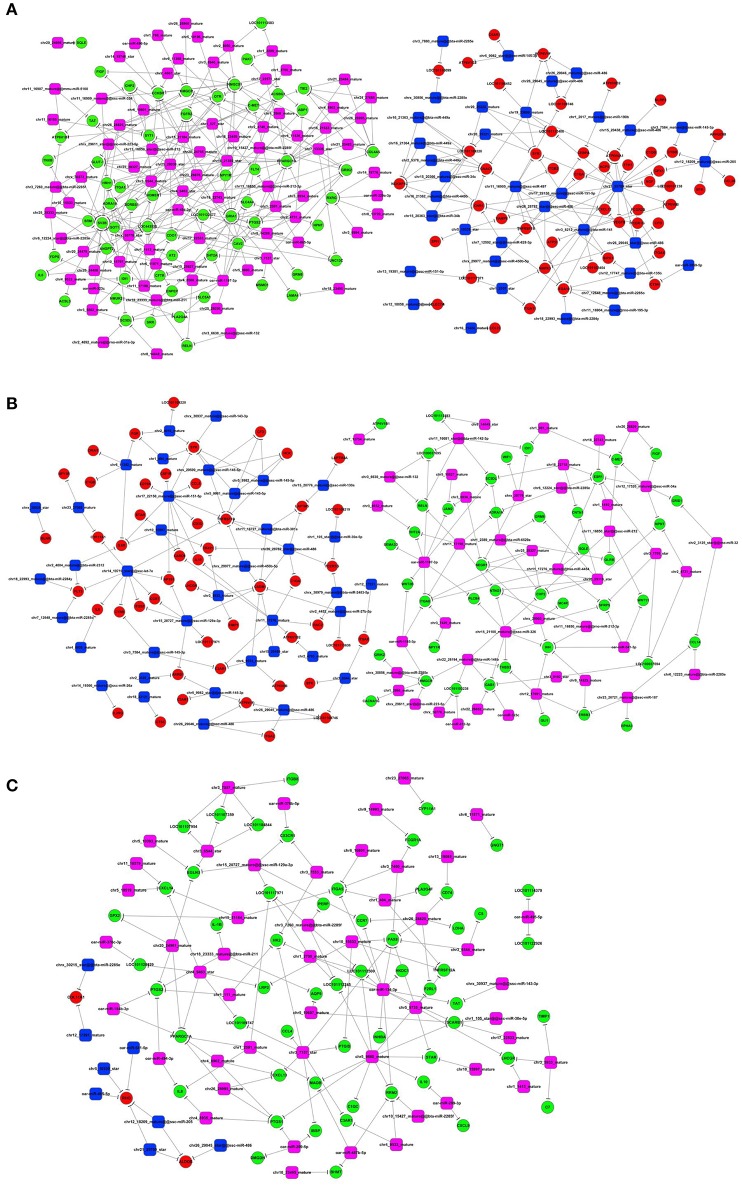
Integrative analysis of the differentially expressed genes and the miRNAs. **(A–C)** Construction of miRNA-mRNA regulatory networks for the different groups. Green indicates the down-regulated mRNA, and red indicates the up-regulated mRNA. Blue indicates the down-regulated miRNA, and purple indicates the up-regulated miRNA.

To investigate whether miRNAs play a key role in sheep fecundity, the small RNAs were extracted from the three sheep groups and were sequenced using the Illumina platform. A systematic analysis enabled the identification of 132, 98, and 100 differentially expressed and previously unreported miRNAs in the Han BB vs. Dorset, the Han ++ vs. Dorset and the Han BB vs. Han ++ comparison groups, respectively. In addition to identifying novel miRNA, we also identified previously reported miRNAs. A total of 10, 6, and 16 known miRNAs were considered to be differentially expressed in the Han BB vs. Dorset, the Han BB vs. Han ++, and the Han ++ vs. Dorset comparison groups, respectively. Next, the Miranda program was used to predict the putative targets of the differentially expressed miRNAs. To study whether the miRNAs play a regulatory role in sheep fecundity, a miRNA-mRNA regulatory network was constructed (Figure 2).

### Gene co-expression network analysis

**Figure 3:**
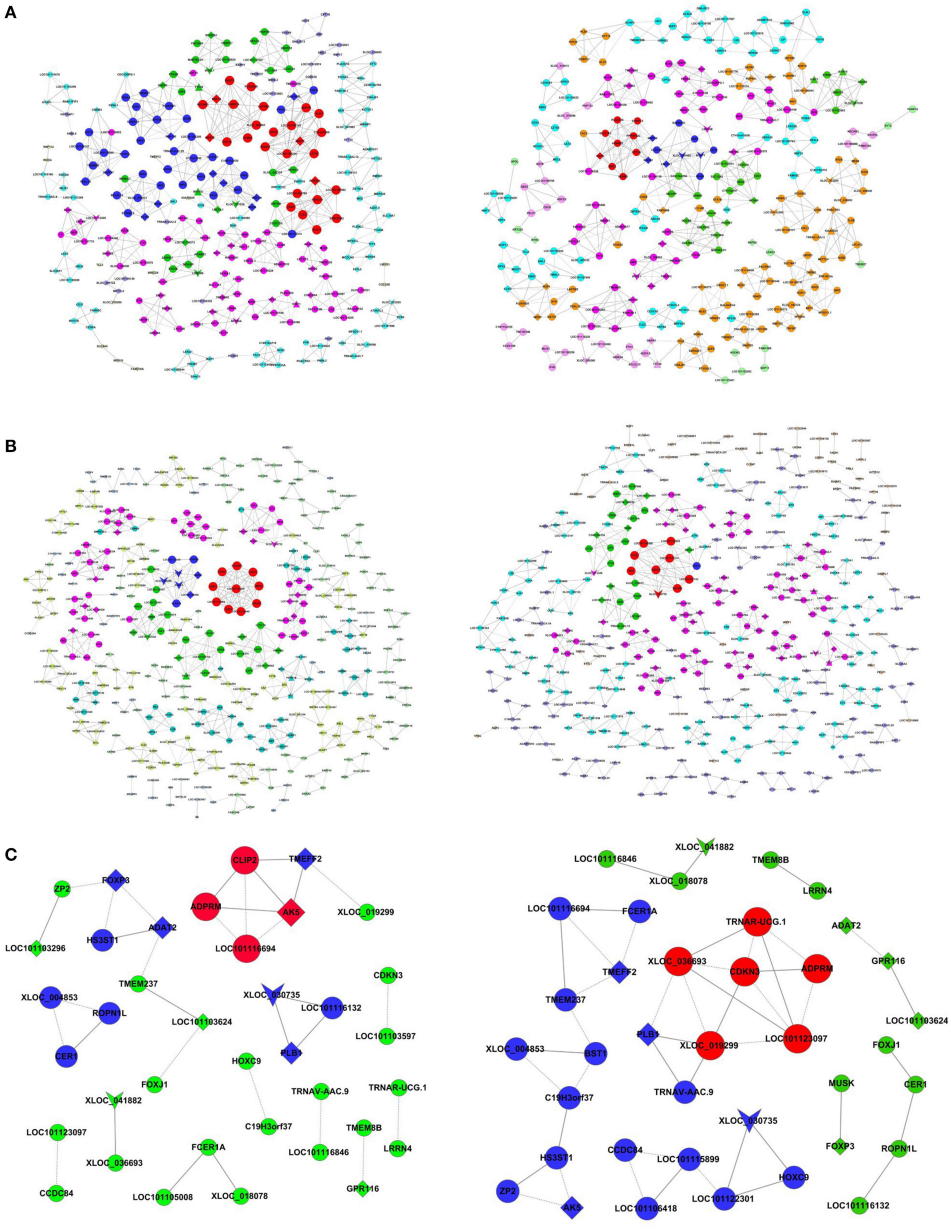
Co-expression networks of the fecundity-associated genes and the co-regulated lncRNAs. **(A–C)** illustrates the networks derived from the samples (left) and the control group (right). **(A)** Han BB vs. Dorset; **(B)** Han ++ vs. Dorset; **(C)** Han BB vs. Han ++. Han BB, Small Tail Han ewes with the genotype BB (HanBB) and genotype ++ (Han ++); Dorset, Dorset sheep. The inverted triangle denotes the lncRNA, and the triangle denotes the methylated genes encoding the lncRNA. The circle denotes the mRNA, and the diamond denotes the methylated genes encoding the mRNA. The nodes with the same color represent the genes with a similar co-expressing ability. The node size represents the node degree.

Methylated coding-non-coding gene co-expression networks for the Han sheep and the control were constructed with all of the genes encoding the methylated DEGs and the differentially expressed lncRNAs identified in this study. The methlyated lncRNA-mRNA pairs with Pearson correlation coefficients that were equal to or >0.99 were selected to construct the co-expression network (Figure 3). The number of nodes was similar in each comparison, but there was different in degree of nodes (Tables S7–S9). In the Han BB vs. Dorset comparison, the lncRNAs tetratricopeptide repeat domain 26 (TTC26) and myosin heavy chain 15 (MYH15) had the largest degree, but the degree of these lncRNAs was different between the Han BB and Dorset groups. Similarly, the lncRNA neuronal tyrosine-phosphorylated phosphoinositide-3-kinase adaptor 1 (NYAP1) had the largest degree in the Han ++ vs. Dorset comparison. However, none of the methylated lncRNAs were co-expressed with either an mRNA or a methylated mRNA in the Han BB vs. Han ++ comparison. These methylated lncRNAs may play a vital regulatory role in sheep breeding.

**Figure 4:**
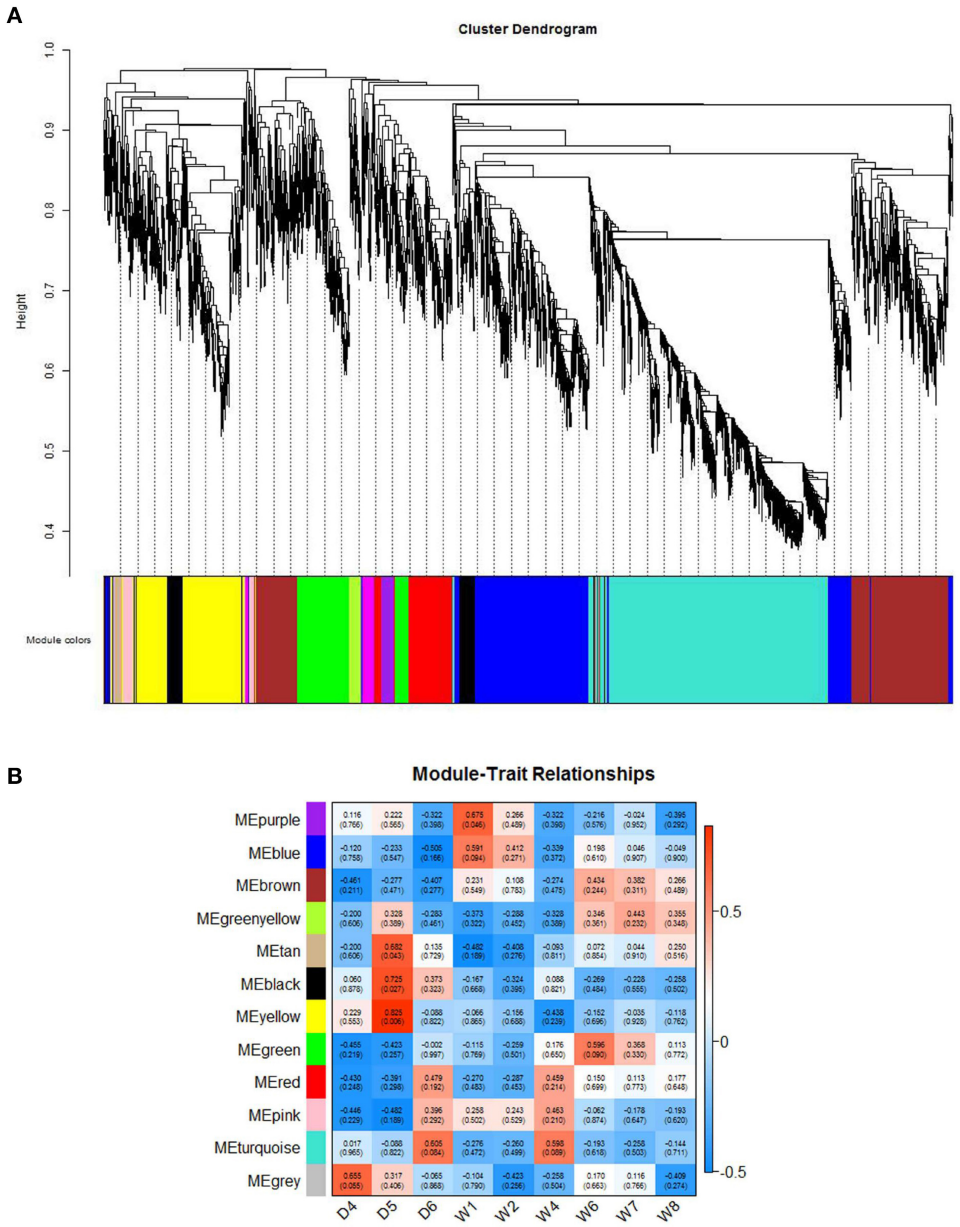
Network analysis of sheep fecundity. **(A)** A hierarchical cluster tree showing the co-expression modules identified using the WGCNA. The modules correspond to the branches and are denoted by colors. **(B)** Heatmap reporting the correlations and the corresponding *p*-values between the modules and the sheep groups. The rows indicate the reported modules, and the columns represent the different sheep groups. The color indicates the level of correlation between the gene co-expression in the sheep group.

To further understand the co-expression relationships between the genes that were significantly correlated to sheep breeding at a systems level, we applied the weighted gene co-expression network analysis (WGCNA), which analyzed the RNA-Seq datasets using a gene co-expression network analysis approach that is an unsupervised and unbiased analysis. The WGCNA identified distinct co-expression modules with respect to clusters of the correlated genes or lncRNAs (Figure 4A). Notably, 8 out of the 13 co-expression modules were significant, and these modules consist of genes that correspond to sheep breeding (Figure 4B). These modules were labeled by color. For example, the black and yellow modules correlated with a high significance with the Dorset samples, but the purple module was strongly correlated with Han sheep sample. Additionally, to determine the GO terms that were enriched in the genes of all of the modules, DAVID was applied. Table 2 shows the top 3 GO biological processes of the modules that are associated with sheep breeding. Importantly, the hub genes and the lncRNAs in the eight modules that might be useful for sheep breeding are listed in Table 3.

**Table 2:** The top 3 GO terms of the methylated genes in each co-expression module.

**Module**	**Top GO terms**	**No. of genes**	***P*-value**	**Enrichment**
Black	GO:0014009: glial cell proliferation	133	0.001042794	59.45112782
	GO:0021782: glial cell development		0.001451638	47.56090226
	GO:0008366: axon ensheathment		0.002460429	33.97207304
Purple	GO:0007276: gamete generation	47	0.002463609	30.58800774
	GO:0034504: protein localization to nucleus		0.00334601	25.88216039
	GO:0006956: complement activation		0.00462532	21.70761839
Blue	GO:0019083: viral transcription	487	3.0267E-27	8.97024289
	GO:0006415: translational termination		1.28824E-26	8.636244484
	GO:0006614: SRP-dependent cotranslational protein targeting to membrane		9.96114E-26	7.961953088
Tan	GO:0051926: negative regulation of calcium ion transport	30	0.000257168	105.4266667
	GO:0060348: bone development		0.001998445	34.00860215
	GO:0007005: mitochondrion organization		0.003218648	26.35666667
Yellow	GO:0035456: response to interferon-beta	342	0.000126457	20.55100715
	GO:0006351: transcription, DNA-templated		0.000172979	1.650612677
	GO:0035455: response to interferon-alpha		0.000233256	16.81446039
Green	GO:0035082: axoneme assembly	169	4.11223E-06	53.47083686
	GO:0001539: cilium or flagellum-dependent cell motility		6.38066E-06	23.39349112
	GO:0030030: cell projection organization		7.99659E-05	6.290686689
Turquoise	GO:0006811: ion transport	540	5.12741E-06	2.129041653
	GO:0007186: G-protein coupled receptor signaling pathway		9.44239E-06	1.764167782
	GO:0007275: multicellular organismal development		6.79523E-05	1.76967006

**Table 3:** The hub genes and the lncRNAs of the 7 modules.

**Module**	**Hub gene name**	**Gene type**	**Description**
Black	AARD	mRNA	Alanine and arginine rich domain containing protein
	LOC101115876	lncRNA	Glycine cleavage system H protein, mitochondrial-like
Purple	ABTB1	mRNA	Ankyrin repeat and BTB (POZ) domain containing 1
	LOC101102687	lncRNA	Ribosomal protein L6 pseudogene
Blue	ABCA9	mRNA	ATP-binding cassette sub-family A member 9
	LOC101102249	lncRNA	Methylthioadenosine phosphorylase pseudogene
Tan	AAMDC	mRNA	Mth938 domain-containing protein
	LOC101108784	lncRNA	60S ribosomal protein L10-like
Yellow	ABCC1	mRNA	Multidrug resistance-associated protein 1
	LOC101102899	lncRNA	Zinc finger protein 709-like
Green	AASDH	mRNA	cDNA FLJ51442, highly similar to Homo sapiens 2-aminoadipic 6-semialdehyde dehydrogenase
	LOC101102556	lncRNA	Putative elongation factor 1-alpha-like 3-like
Turquoise	AADACL2	mRNA	Arylacetamide deacetylase-like 2
	LOC101101914	lncRNA	Small nuclear ribonucleoprotein-associated protein N-like

### Real-time quantitative PCR validation

**Figure 5:**
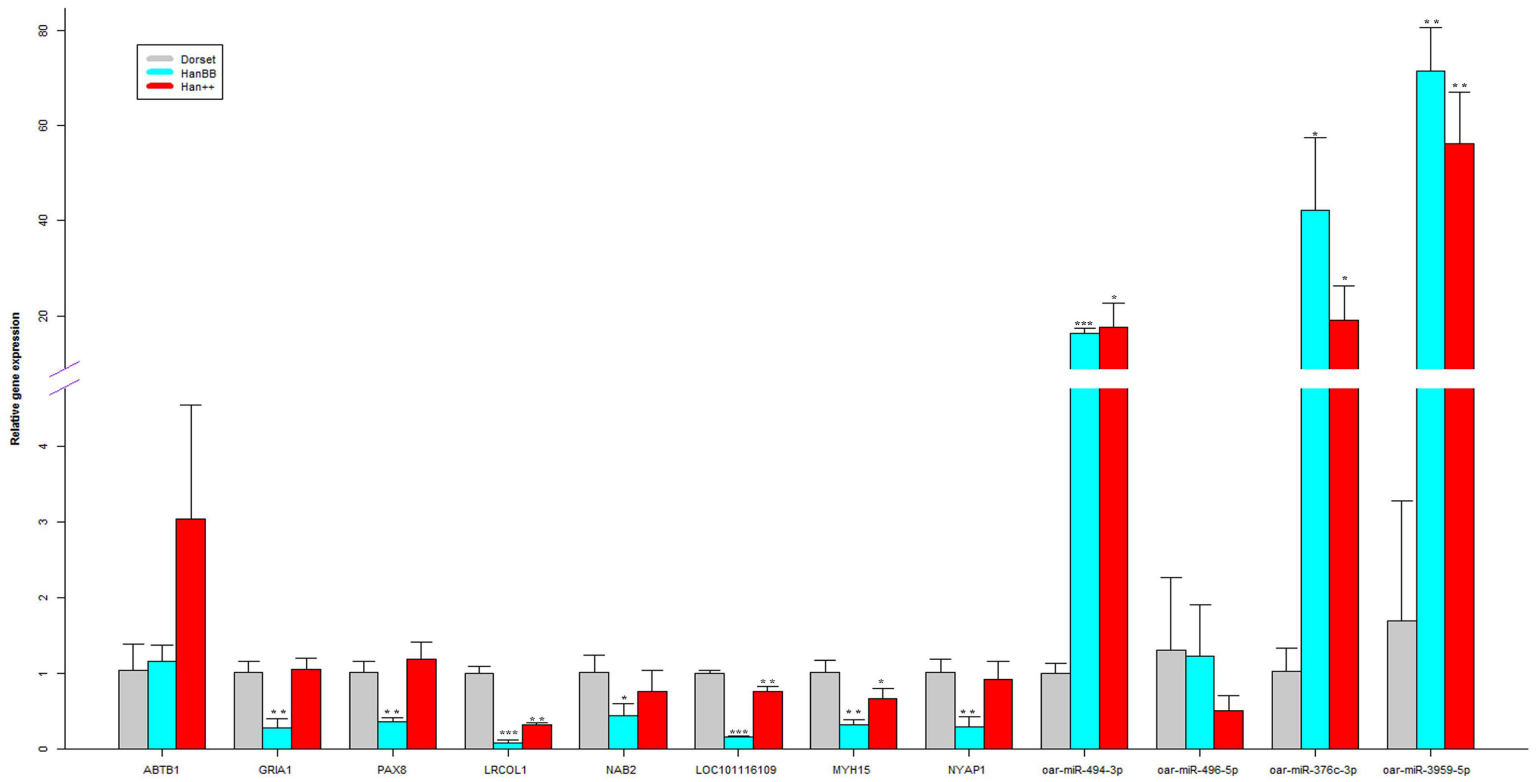
Validation of the RNA-Seq data by real-time quantitative PCR. The relative expression levels of each mRNA and lncRNA were normalized to 18S rRNA, and each miRNA was normalized to 5S rRNA. ^*^p < 0.05, ^**^p < 0.01, and ^***^p < 0.001, respectively.

To validate the RNA-Seq data, 6 methylated genes encoding differentially expressed mRNAs, 2 methylated genes encoding differentially expressed lncRNAs and 4 differentially expressed miRNAs were randomly selected and analyzed via real-time PCR (Figure 5, Table S10). For the methylated genes encoding the differentially expressed mRNAs, the results demonstrated that ankyrin repeat and BTB (POZ) domain containing 1 (ABTB1) were up-regulated and that the glutamate receptor, ionotropic, AMPA 1 (GRIA1), paired box 8 (PAX8), leucine rich colipase-like 1 (LRCOL1), NGFI-A binding protein 2 (NAB2), and LOC101116109 genes were down-regulated in the Han sheep group in comparison to the Dorset group. Additionally, with regard to the lncRNAs, MYH15, and NYAP1 were down-regulated in the Han BB sheep group in comparison to the Dorset group. For the miRNAs, the expression of oar-miR-494-3p, oar-miR-496-5p, oar-miR-376c-3p, and oar-miR-3959-5p showed statistically significant differences between the Han sheep and the Dorset group (*p* < 0.05). These results validated the RNA-Seq data, providing reassurance that the above findings can be trusted.

## Discussion

Sheep fecundity is vital for agriculture but varies in sheep with different genetic backgrounds. Here, we conducted genome-wide analyses to identify miRNAs and methylated genes encoding lncRNAs that were differentially expressed in Dorset sheep and Han BB and Han ++ sheep using RNA-Seq technology. The study is an extension of our previous study, in which we examined the regulatory relationships between lncRNAs, miRNA, and mRNAs in the same 3 groups of sheep. In the current study, we specifically sought to find a relationship between the methylated genes encoding the DEGs and the differentially expressed lncRNAs by generating a co-expression network.

It is already known that the Han BB ewes have a mutation in the BMPR1B gene and display higher ovulation rates, larger oocyte diameters at the earlier stages of follicular development and smaller follicle diameters at ovulation compared to the Han ++ sheep (Reader et al., 2012). The BMPR1B pathway is a well-studied pathway related to sheep fecundity, but even with such studies much still remains unknown about sheep fecundity. This study aimed to take a novel approach to looking at gene dysregulation in sheep with different fecundity.

Through the GO and KEGG analyses, we found that the differentially expressed genes in the Han BB sheep, which have the highest proliferacy of the 3 groups, were involved in metabolism, which was in line with a previous study from our group and from another group (Quintero-Elisea et al., 2011; Miao et al., 2015b). Taking this analysis a step further, we identified not only the methylated DEGs between the Han sheep and the Dorset sheep, but also the differentially expressed miRNAs. One of the miRNAs, rno-miR-34b-3p, is specifically expressed in rat testes (Linsen et al., 2010). In the current study, this miRNA showed differential expression in the Han BB sheep compared with the Dorset group, indicating that this miRNA might distinguish between these 2 breeds and could be associated with sheep fecundity.

Specifically, we utilized an integrative analysis of the mRNAs and miRNAs. Here, we found a number of miRNA-mRNA pairs that might contribute to our understanding the genetic regulation relationship between the regulators. For instance, there was a differential expression of oar-miR-134-3p in the Han ++ sheep in comparison with the Dorset sheep. It is known that oar-miR-134-3p is dysregulated in sheep skeletal muscle (Caiment et al., 2010). Interestingly, our study indicated that PAX8 was negatively correlated to oar-miR-134-3p and, furthermore, was regulated by several other miRNAs. The follicular cells of the thyroid gland require the PAX8 gene, and the rearrangement of PAX8 might be related to thyroid tumors, which is in accordance with the representation of the pathway that PAX8 was mainly involved in thyroid hormone synthesis and thyroid cancer pathways (Mansouri et al., 1998; Nikiforova, 2003). Thyroid hormone synthesis plays a key role in exerting direct stimulatory effects on granulose cell functions, such as steroidogenic enzyme induction (Maruo et al., 1987). Steroidogenic enzymes are associated with the production of ovarian steroids, including 3β-hydroxysteroid dehydrogenase and aromatase, which play a key role in animal prolificacy (Takayama et al., 1996). Here, we found that PAX8 was down-regulated in Han BB sheep compared with Dorset. Taken together, these data suggest that differentially expressed oar-miR-134-3p may play an important role in sheep breeding by regulating thyroid hormone synthesis through PAX8. This finding is worth further exploration and offers a novel pathway that might be important in understanding sheep proliferacy.

Data from our previous study, which provided the basis for this study, and others studies which provided increasing evidence, suggest that lncRNAs play an important role in development (Bao et al., 2013; Li et al., 2015; Miao et al., 2016d). However, genome-wide analyses of the lncRNAs associated with sheep breeding are lacking. Notably, DNA methylation, which is thought to be one of the main anomalous alterations in development, regulates hundreds of thousands of genes. However, there are few studies about the DNA methylation of coding and non-coding genes related to sheep fecundity. Epigenetic silencing of BMPR1B was reported in a subset of glioblastoma cells. A deregulated BMPR1B pathway in a subset of glioblastoma cells contributes to their tumorigenicity by desensitizing the cells to normal differentiation cues and by converting cytostatic signals into pro-proliferative signals (Lee et al., 2008). Clearly, epigenetic regulation of tumor cells through a gene that plays a huge role in sheep fecundity suggests that this type of regulation might also occur in the ovaries of sheep. In the current study, we identified several methylated genes that encode the DEGs and lncRNAs that showed differential expression in the Han sheep in comparison with the Dorset sheep using MeDIP-Seq. We carried out a comprehensive analysis of the methylated genes in relation to the mRNA and lncRNA co-expressions between the high fecundity (Han) and low fecundity (Dorset) sheep in order to identify genes that might distinguish these 2 breeds. Then, a WGCNA was applied to explore the co-expression networks between these 2 sheep breeds, and thus, we identified 8 significant modules associated with the Dorset and Han sheep groups. Our results provide insights for researchers aiming to better understand the mechanism of sheep fecundity and might aid in the selection of sheep with high fecundity.

Compared with the low-fecundity Dorset sheep, 73 methylated genes encoding the DEGs and the differentially expressed lncRNAs were identified in the Han BB group, which has the highest fecundity, and 129 were identified in the Han ++ group (the fecundity rate falls between the Dorset and Han BB), suggesting that DNA methylation is a common regulator of coding and non-coding genes that define these 2 breeds of sheep. As expected, the methylated genes encoding the DEGs in the Han ++ and Han BB groups were significantly involved in the GO terms related to metabolic process, development and cell differentiation, which were important for sheep reproduction. Interestingly, a previous study demonstrated that the regulation of DNA methylation is more vital for regulating lncRNAs compared with protein coding gene expression in cancer development (Liao et al., 2015). Taken together, we hypothesize that the methylated genes encoding the DEGs and the differentially expressed lncRNAs exert an equally important function in sheep breeding.

An integrated analysis of the methylated genes encoding the DEGs and the differentially expressed lncRNAs identified from the Han BB sheep revealed that the lncRNA TTC26 co-expressed with the gene ring finger protein 13 (RNF13), which is demonstrated to be important in cell proliferation (Jin et al., 2011). More recently, the lncRNA MYH15 encodes a novel myosin in mammalian skeletal muscles and its co-expressed gene, adenosine deaminase, tRNA-specific 2 (ADAT2), is important for RNA editing (Macbeth et al., 2005; Rossi et al., 2010). Our GO functional enrichment analysis showed that MYH15 was obviously involved in tight junction formation, which was previously indicated to play a key role in the regulation of ovarian follicle development and spermatogenesis in zebrafish (Clelland and Kelly, 2010). In addition, for the HanBB sheep, the mean follicular and oocyte diameter of the type 1 follicles is larger, the mitochondria, smooth endoplasmic reticulum and ribosomes have a greater volume, and the surface area of the junctions with the granulosa cells is greater compared with Han++ sheep (Reader et al., 2012). The genes identified in this study that are related to tight junction formation might also be related to the BMPR1B mutation, which is hypothesis that deserves further attention.

Quantitation of the expression by real-time PCR analysis of these lncRNAs in the three sheep groups revealed that TTC26 and MYH15 were down-regulated, suggesting that the methylation of the gene might repress the expression of these lncRNAs. As a result, the methylated genes encoding these lncRNAs might contribute to our further understand of the genetic mechanism associated with sheep breeding.

In addition, the significant modules associated with sheep breeding were also mined using a WGCNA. As shown in Figure 4, the modules purple and blue were associated with the Han BB group. Specifically, the GO enrichment of the genes in module purple was mainly involved in gamete generation, which was an important process for breeding. Furthermore, the hub gene of the module in purple was ABTB1 mediating the phosphatase and tensin homolog (PTEN) growth-suppressive signaling pathway, which suppresses tumor cell proliferation and migration (Unoki and Nakamura, 2001). More importantly, the methylation of ABTB1 down-regulates or even silences gene expression, and as a result, we hypothesize that methylated ABTB1 may play an important role in sheep breeding, contributing to cell generation and proliferation.

Taken together, this study provides an in-depth view into the methylation of genes encoding the trancriptome of 2 breeds of sheep ovaries, which is an extension of our previous findings. Previously, we provided the first elucidation of sheep fecundity-related miRNAs and lncRNAs (Miao et al., 2016e). Here, we made several important observations. First, in addition to the miRNAs analyzed in our previous study, the methylation of genes encoding differentially expressed mRNAs and lncRNAs was analyzed to study differences between these 2 breeds that might be fecundity-associated molecules. In addition, our previous study provided us with co-expression relationships between the mRNAs, lncRNAs, and miRNAs, which were detected to present a regulatory mechanism of sheep fecundity. The current results suggested that miRNAs might play a key role in sheep prolificacy by regulating target genes related to thyroid hormone synthesis and that methylated genes encoding lncRNAs might be associated with tight junction formation, which could contribute to the high breeding rate in the Han sheep and might be related to the BMPR1B mutation. These findings contribute to a deeper understanding of the differences between Han and Dorset sheep and might be related to sheep prolificacy. We hope that this study can provide the framework for future research to explore differences in miRNAs, lncRNAs, and methylated genes between sheep breeds with different fecundity rates and with the BMPR1B mutation.

## Accession number

The accession number GSE107829 for DNA-Seq data. https://www.ncbi.nlm.nih.gov/geo/query/acc.cgi? acc=GSE107829. The accession number GSE107935 for RNA-Seq data. https://www.ncbi.nlm.nih.gov/geo/query/acc.cgi?acc=GSE107935..

## Author contributions

XM conceived, designed and performed the experiments and wrote the paper; QL, HZ and XQ performed the experiments. All of the authors read and approved the final manuscript.

### Conflict of interest statement

The authors declare that the research was conducted in the absence of any commercial or financial relationships that could be construed as a potential conflict of interest.
