# Supplementary figures and images for: An Integrated Analysis of miRNAs and Methylated Genes Encoding mRNAs and lncRNAs in Sheep Breeds with Different Fecundity
Source: Front Physiol. 2017 Dec 15;8:1049. doi: 10.3389/fphys.2017.01049 (PMC5736872; doi:10.3389/fphys.2017.01049)

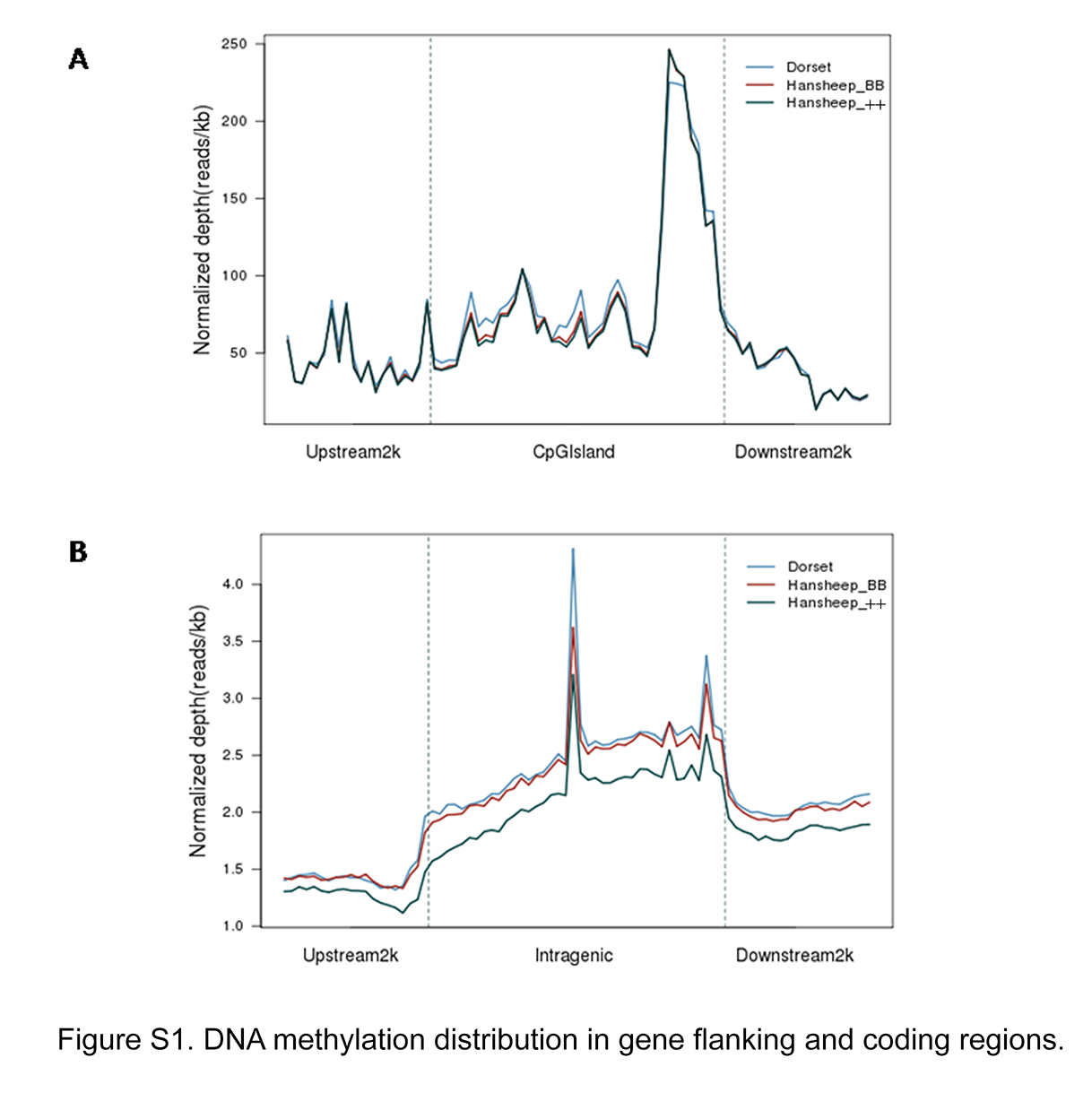

Supplement: Supplementary file 11 [file Image1.TIF]

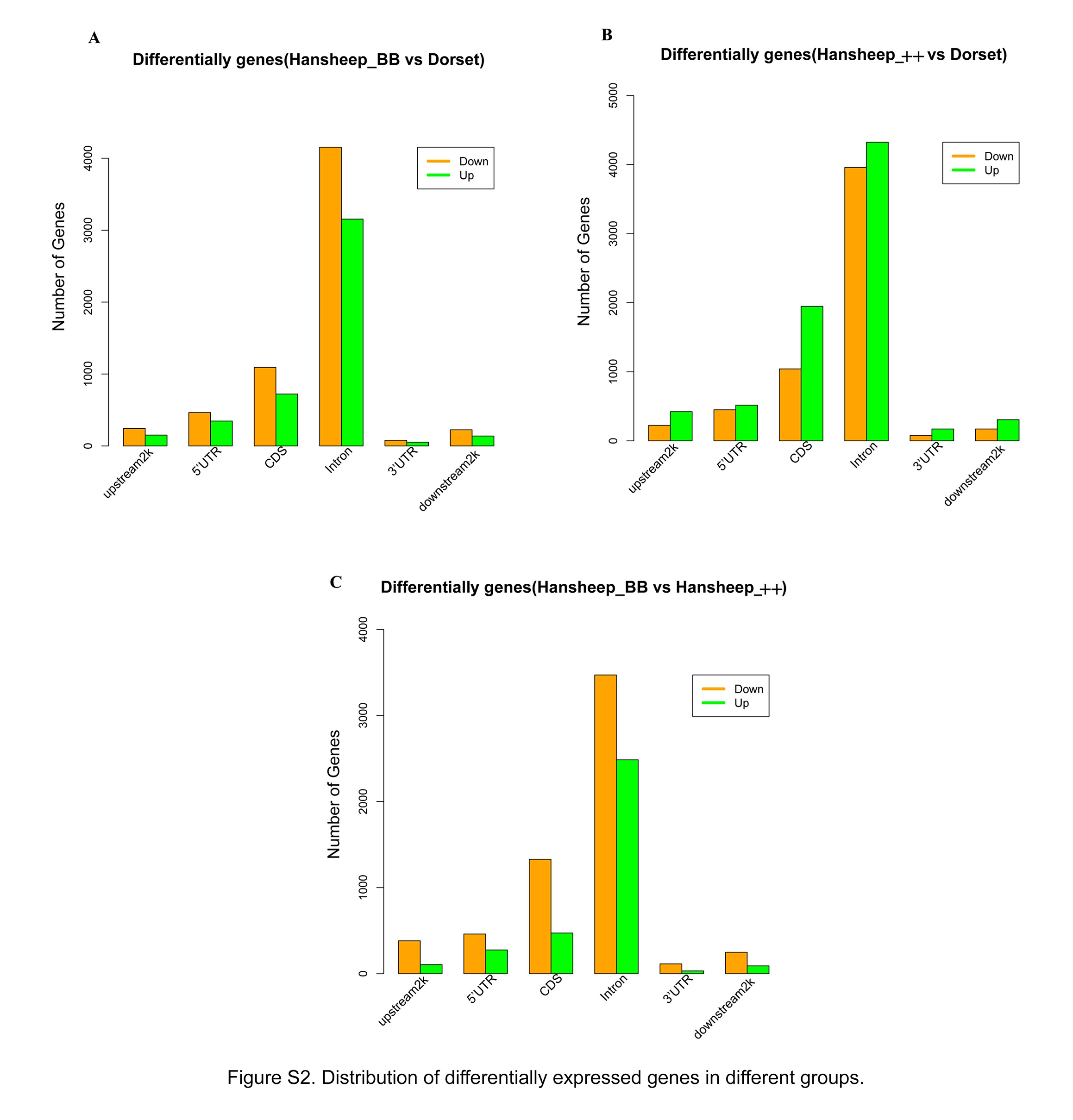

Supplement: Supplementary file 12 [file Image2.TIF]
